# Socioeconomic status and stroke severity: Understanding indirect effects via risk factors and stroke prevention using innovative statistical methods for mediation analysis

**DOI:** 10.1371/journal.pone.0270533

**Published:** 2022-06-24

**Authors:** Anita Lindmark, Marie Eriksson, David Darehed

**Affiliations:** 1 Department of Statistics, Umeå School of Business, Economics and Statistics, Umeå University, Umeå, Sweden; 2 Department of Public Health and Clinical Medicine, Sunderby Research Unit, Umeå University, Sweden; Universita degli Studi Magna Graecia di Catanzaro, ITALY

## Abstract

**Background:**

Those with low socioeconomic status have an increased risk of stroke, more severe strokes, reduced access to treatment, and more adverse outcomes after stroke. The question is why these differences are present. In this study we investigate to which extent the association between low socioeconomic status and stroke severity can be explained by differences in risk factors and stroke prevention drugs.

**Methods:**

The study included 86 316 patients registered with an ischemic stroke in the Swedish Stroke Register (Riksstroke) 2012–2016. Data on socioeconomic status was retrieved from the Longitudinal integrated database for health insurance and labour market studies (LISA) by individual linkage. We used education level as proxy for socioeconomic status, with primary school education classified as low education. Stroke severity was measured using the Reaction Level Scale, with values above 1 classified as severe strokes. To investigate the pathways via risk factors and stroke prevention drugs we performed a mediation analysis estimating indirect and direct effects.

**Results:**

Low education was associated with an excess risk of a severe stroke compared to mid/high education (absolute risk difference 1.4%, 95% CI: 1.0%-1.8%), adjusting for confounders. Of this association 28.5% was an indirect effect via risk factors (absolute risk difference 0.4%, 95% CI: 0.3%-0.5%), while the indirect effect via stroke prevention drugs was negligible.

**Conclusion:**

Almost one third of the association between low education and severe stroke was explained by risk factors, and clinical effort should be taken to reduce these risk factors to decrease stroke severity among those with low socioeconomic status.

## Background

Stroke is the third leading cause of death and disability combined, and the second leading cause of death worldwide [1]. Strokes are mainly classified as either ischemic, caused by a clot in a cerebral blood vessel, or hemorrhagic, caused by a bleed in the brain. Almost half of all stroke-related mortality may be due to modifiable risk factors, where only 10 risk factors account for around 90% of the total modifiable risk for stroke [2, 3]. Differences in age-standardized rates of stroke disability and mortality that are attributable to modifiable risk factors are seen between countries of different income levels, where those with high income are at less risk than those with lower income [4].

Even within countries, socioeconomic status (SES) is an important factor when considering stroke risk, care and outcomes. SES is defined as an individual’s economic and social position relative to others, typically measured by education, occupation and income [5]. Differences in stroke risk, severity and outcomes depending on SES are well established, where those with lower SES are at greater risk of a stroke and suffer more adverse outcomes [3, 6–10]. Even in Sweden, a country with relatively limited income inequalities, and publicly financed health care, SES differences in stroke care are present where those with lower SES have poorer access to stroke unit care, acute treatment and secondary prevention [11–13].

The underlying reasons for these differences remain largely unclear, and more important yet, how can they be prevented. Attempts to explain SES differences in stroke have often included adjusting for possible intermediate factors and investigating to which extent this attenuates the effects of SES. One example is a large meta-analysis which found that modifiable vascular risk factors accounted for around 50% of the increased risk of stroke for those with low SES [14]. It has been argued that this risk may be higher since patients with lower SES are more likely to be exposed to more risk factors, and risk factors in combination may have a multiplicative effect [15].

An alternative to adjustment for possible intermediate factors is performing a mediation analysis where the effect of SES is separated into indirect and direct effects [16]. Indirect effect(s) then capture the effect of SES on the outcome that operates through some intermediate variable(s) (mediators) of interest (e.g. risk factors) while the direct effect captures the effect of SES on the outcome that does not operate through the intermediate variable(s). This separation can then give an idea of e.g. how much of the SES difference that would remain if we could intervene on the mediators.

Previous studies suggest that patients with low SES have more severe strokes than patients with high SES [6, 8, 17], and that a substantial part of SES differences in survival up to 3 months after stroke could be explained by differences in stroke severity [18]. In this nationwide, register-based study we investigate the extent to which differences in modifiable risk factors and access to stroke prevention drugs explain the relationship between low SES and stroke severity.

## Methods

Requests to access the data set from qualified researchers trained in human subject confidentiality protocols may be sent to the Swedish Stroke Register (Riksstroke) at riksstroke@regionvasterbotten.se.

### Setting

In Sweden, primary and secondary healthcare is provided to the population through 21 different regions. Healthcare is mostly tax funded, apart from a small co-payment made by the patient. Acute stroke care is provided at 72 hospitals across the country [19].

### Study design

This retrospective register-based cohort study included all adult patients registered in Riksstroke, a quality register for hospital stroke care, with an acute ischemic stroke (*International Classification of Diseases*, *Tenth Revision*: I63) in Sweden between 2012 and 2016. Riksstroke retrieved the study population. All 72 hospitals that provide acute stroke care register patients in Riksstroke and the register has been shown to cover up to 96% of all patients treated in hospital for acute stroke, meaning that the risk of selection bias is minimal [19]. Annually there are approximately 20 000 strokes registered, with a decreasing trend over time [19]. The quality register holds information on temporal data (stroke onset time, admission time etc.), patient characteristics (sex, age, cardiovascular risk factors, stroke severity, primary prevention etc.), acute care, secondary prevention and outcomes (mortality, functional outcomes etc.). At the acute stage information is collected by hospital staff. Patients and next of kin are informed about the registration and aim of the register and their right to decline participation (opt-out consent). Data are automatically checked upon entry and data validity is evaluated continuously. Riksstroke data were linked with data from the Longitudinal integrated database for health insurance and labour market studies (LISA) managed by Statistics Sweden, from which data on socioeconomic status was obtained. The linkage was performed through the personal identity numbers (Swedish national identification numbers) of the patients and was done by Statistics Sweden. Ethical approval was obtained from the regional ethics review board in Umeå, Sweden (reference number 2017/184-31). Results were reported according to the Reporting of Studies Conducted Using Observational Routinely Collected Health Data (RECORD) Statement (S1 Table).

### Statistical methods

#### Variables

**Fig 1:**
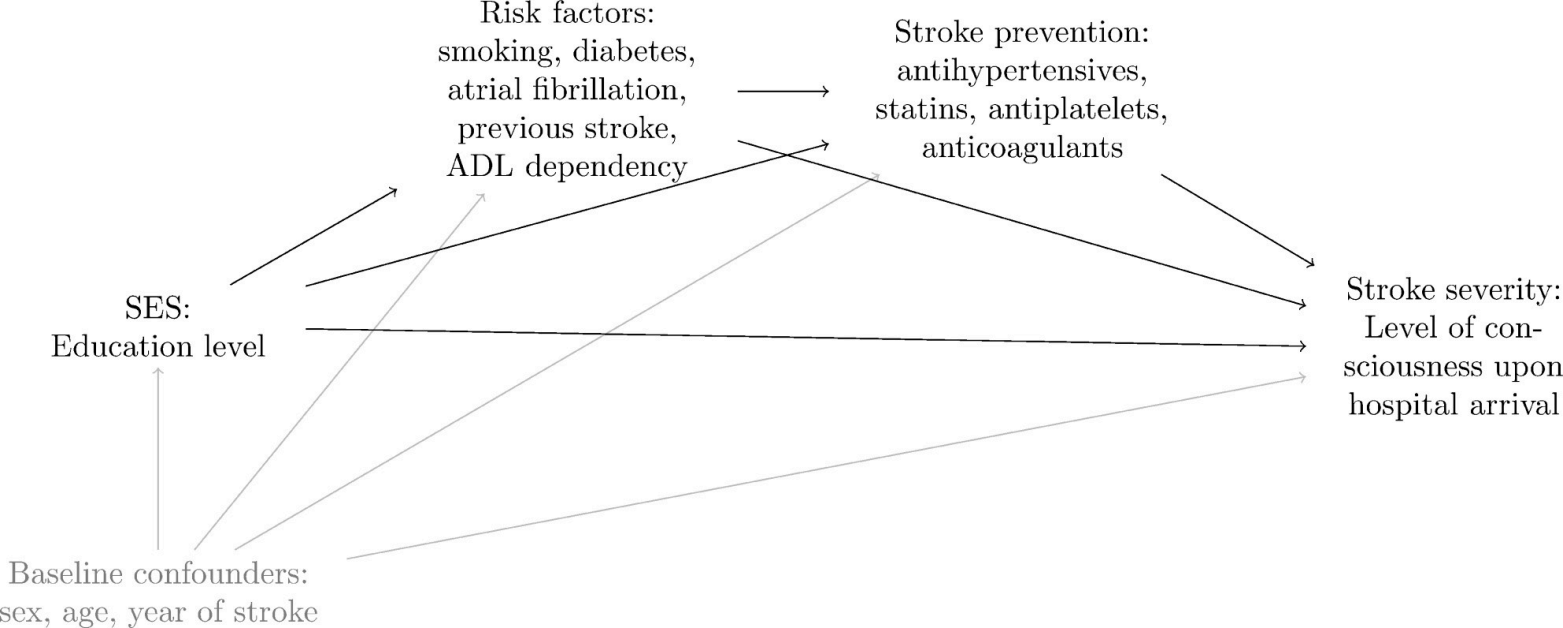
DAG illustrating the hypothesized relationships between the variables in the study.

For our main exposure, low SES, we used low education as proxy. The rationale behind using education level rather than e.g. occupation or income as the measure of SES is that it tends to be more stable across the life course and that education is related to both material and non-material resources [20]. We defined the exposure as having low education (only primary school) vs. having mid/high education (secondary school/university). For our outcome, stroke severity, we used level of consciousness upon arrival to the hospital, measured using the Reaction Level Scale as proxy. An RLS point of 1 (alert) versus RLS points of 2–8 (lowered consciousness) were used. We used the directed acyclic graph (DAG) methodology to create an illustration of the relationship between education level and stroke severity, including possible confounders and mediators [21]. We sorted variables into three groups; “baseline confounders” (sex, age, year of stroke), “risk factors” (smoking, diabetes, atrial fibrillation, previous stroke, activities in daily living (ADL) dependency (defined as inability to move around indoors, manage dressing, and/or using the bathroom without assistance) at time of stroke) and “stroke prevention drugs” (antihypertensives, statins, antiplatelets, anticoagulants), with the latter two hypothesized to lie on the pathway between education and stroke severity. The variables screened for inclusion were based on clinical experience, but ultimately decided upon depending on availability in the register. See Fig 1 for the DAG and all included variables.

#### Mediation analysis

We used a causal inference approach to mediation which, compared to the traditional approach [22], has the advantages that direct and indirect effects can be defined more generally, that the assumptions required to estimate effects from data are explicit, and that effects can be estimated using a range of different methods [23].

Different versions of direct and indirect effects have been suggested depending on the aim of the causal mediation analysis [23]. Here we estimate so-called *interventional disparity effects* [24] with a focus on how the disparity in stroke severity between patients with low education and patients with mid/high education might change if we were to intervene on the mediators in the “risk factor” and “primary prevention” pathways. We separated the total association between education and stroke severity into direct and indirect components. The direct component is represented by the *interventional disparity direct effect* which corresponds to the extent to which the association between education and stroke severity would remain if the distributions of risk factors and stroke prevention drugs were made to be the same in patients with low education as patients with mid/high education [24]. The *interventional disparity indirect effect* corresponds to the extent by which the stroke severity of patients with low level of education would change had the distributions of their mediators been changed to those of patients with mid/high level of education. We estimate the interventional disparity indirect effects through all mediators taken jointly, as well as through the mediators in the “risk factor” and “stroke prevention drugs” pathways separately and through the dependence between the mediators in the two pathways.

To gain an idea of the relations in Fig 1 we estimated the associations between the exposure low education and the outcome stroke severity and each of the mediators, as well as the associations between each of the mediators and stroke severity using separate logistic regression models with and without adjustment for other covariates. Descriptive statistics are presented for age categories, but the analysis models include age and age squared as continuous covariates.

The total association and direct and indirect effects were estimated through Monte Carlo simulation based on logistic regression models for the outcome, given exposure, mediators and confounders, and mediators, given exposure and confounders [24–26]. The general estimation procedure is described in detail elsewhere [24]. All 2-way interactions and age squared were included in the models to make them flexible, reducing the risk of model misspecification bias [26]. The robustness of the results to alternative model specifications was checked with analyses based on models with only main effects. Standard errors of the effects were estimated through bootstrap.

The estimation procedure combines Monte Carlo simulation and bootstrap and is thus very computer intensive. For this reason, we opted to perform complete case analyses, meaning that patients with missing information on any variable were excluded, rather than adding multiple imputation steps to the procedure. A sensitivity analysis was performed, where the effects were re-estimated based on an imputed data set (single stochastic imputation using chained equations with 10 burn in iterations) [27].

All analyses were performed in R [28]. The code is available from the corresponding author upon request.

## Results

### Baseline data

We identified 101 261 patients with ischemic stroke in 2012–2016, of which 86 316 had complete records (i.e. no missing values) on all study variables and were included in this study. The total proportion of patients with any missing value was 14.8% with the largest proportions of missing values observed for smoking (9.5%) and ADL-dependency at baseline (3%), with less than 2% missing values for all other variables (S2 Table).

Of the patients with complete records 47.4% were female (Table 1) and the average age was 75.3 years, 43.5% of the patients had low education and 11.7% had lowered consciousness upon hospital arrival. The most prevalent risk factor was atrial fibrillation (28.9%), followed by previous stroke (22.8%) and diabetes (21.5%). The most prescribed stroke preventive drug was antihypertensives (66.5%), followed by antiplatelets (38.9%), statins (29.8%) and anticoagulants (10.1%).

**Table 1:** Patient characteristics, complete records. Number (%).

Variable	n	(%)
***Exposure***:		
**Education**		
**Low (primary school)**	37 565	(43.5)
**Mid/high (secondary school/university)**	48 751	(56.5)
***Outcome***:		
**Level of consciousness**		
**Fully conscious**	76 230	(88.3)
**Lowered**	10 086	(11.7)
***Mediators***:		
**Smoker**	12 622	(14.6)
**Diabetes**	18 565	(21.5)
**Atrial fibrillation**	24 923	(28.9)
**Previous stroke**	19 670	(22.8)
ADL^a^ dependent at baseline	10 333	(12.0)
**Antihypertensive treatment**	57 429	(66.5)
**Statin treatment**	25 684	(29.8)
**Antiplatelet treatment**	33 538	(38.9)
**Anticoagulant treatment**	8716	(10.1)
***Confounders***:		
**Female sex**	40 930	(47.4)
**Year of stroke**		
**2012**	18 933	(21.9)
**2013**	18 354	(21.3)
**2014**	17 563	(20.3)
**2015**	16 321	(18.9)
**2016**	15 145	(17.5)
**Age**		
**18–54**	5430	(6.3)
**55–64**	9666	(11.2)
**65–74**	21 792	(25.2)
**75–84**	27 558	(31.9)
**84+**	21 870	(25.3)

ADL: Activities in daily living.

Low education was associated with an increased the risk of having lowered consciousness upon arrival also after adjustment for confounders (OR: 1.16, 95% CI 1.11–1.21, Table 2). After adjustment low education also increased the risk of all risk factors except for atrial fibrillation (0.98, 0.95–1.02). The biggest increase in risk was seen for smoking, diabetes and ADL-dependency. For stroke prevention drugs, low education increased the chance of receiving prophylactic treatment except for anticoagulants where low education was associated with a decreased chance to receive treatment (0.92, 0.88–0.96).

**Table 2:** Unadjusted and adjusted associations between the exposure, mediators and the outcome estimated through separate logistic regression models.

Associations	Unadjusted OR	95% CI	Adjusted OR^A^	95% CI
***Exposure–outcome model***:				
Low education–severe stroke^b^	1.45	1.40 – 1.52	1.16	1.11 – 1.21
***Exposure–mediator models***:				
**Low education–smoker**	0.88	0.85 – 0.92	1.37	1.31 – 1.43
**Low education–diabetes**	1.21	1.17 – 1.25	1.25	1.20 – 1.29
**Low education–atrial fibrillation**	1.36	1.32 – 1.40	0.98	0.95 – 1.02
**Low education–previous stroke**	1.20	1.16 – 1.23	1.05	1.01 – 1.08
**Low education–ADL dep. at baseline**	1.81	1.74 – 1.89	1.26	1.20 – 1.31
**Low education–antihypertensives**	1.49	1.45 – 1.53	1.16	1.12 – 1.19
**Low education–statins**	1.07	1.04 – 1.10	1.08	1.05 – 1.12
**Low education–antiplatelets**	1.36	1.32 – 1.39	1.09	1.06 – 1.13
**Low education–anticoagulants**	1.06	1.01 – 1.11	0.92	0.88 – 0.96
***Mediator–outcome models***:				
**Smoker–severe stroke**	0.66	0.62 – 0.71	0.98	0.92 – 1.06
**Diabetes–severe stroke**	1.05	1.00 – 1.11	1.11	1.06 – 1.17
**Atrial fibrillation–severe stroke**	2.36	2.26 – 2.46	1.90	1.82 – 1.98
**Previous stroke–severe stroke**	1.38	1.32 – 1.45	1.27	1.21 – 1.34
**ADL dep. at baseline–severe stroke**	3.74	3.56 – 3.92	2.88	2.74 – 3.03
**Antihypertensives–severe stroke**	1.28	1.22 – 1.34	1.02	0.97 – 1.07
**Statins–severe stroke**	0.83	0.79 – 0.87	0.89	0.85 – 0.93
**Antiplatelets–severe stroke**	1.21	1.16 – 1.26	1.01	0.97 – 1.05
**Anticoagulants–severe stroke**	1.17	1.09 – 1.25	1.09	1.02 – 1.16

OR: Odds ratio, ADL: Activities in daily living.

^a^The exposure-outcome and exposure-mediator models adjust for the confounders age, sex and year of stroke. The mediator-outcome models adjust for the confounders and the exposure low education.

^b^Defined by lowered consciousness upon hospital arrival.

All risk factors except for smoking were associated with an increased risk of lowered consciousness (Table 2). The greatest increase was associated with ADL-dependency at baseline (2.88, 2.74–3.03). For stroke prevention drugs the results were more disparate, with small associations between lowered consciousness and antihypertensive treatment and antiplatelets, decreased risk of lowered consciousness for patients with statins (0.89, 0.85–0.93) and increased risk for patients with anticoagulants (1.09, 1.02–1.16).

### Indirect and direct effects of education on stroke severity

Low education was associated with an excess risk of lowered consciousness of 1.4% (95% CI: 1.0%-1.8%) compared to mid/high education, adjusting for confounders (Table 3). The direct effect was 1.0% (0.6%-1.4%), meaning that 71.3% of this association would remain if risk factors and stroke prevention drugs among patients with low education had the same distributions as among patients with mid/high education with the same sex, age and year of stroke. The indirect effect through all mediators taken jointly was 0.4% (0.3%-0.5%), which corresponds to the reduction in risk of severe stroke in patients with low education, if their risk factors and stroke prevention drugs had the same distributions as those of patients with mid/high level of education with the same sex, age and year of stroke.

**Table 3:** Adjusted total association, direct and indirect effects estimated as absolute risk differences (excess risks).

Effect	Absolute risk difference	95% CI	P-value	% of adj. total association
**Adjusted total association:**	1.4%	1.0% – 1.8%	<0.001	
**Direct:**	1.0%	0.6% – 1.4%	<0.001	71.3
**Indirect via:**				
**all mediators**	0.4%	0.3% – 0.5%	<0.001	28.7
**risk factors**	0.4%	0.3% – 0.5%	<0.001	28.5
**stroke prevention drugs**	-0.01%	-0.05% – 0.03%	0.634	-0.6
**dependence between risk factors and stroke prevention drugs**	0.01%	-0.02% – 0.04%	0.375	0.9

Estimates are based on 500 Monte Carlo simulations and standard errors are based on 1000 bootstrap replicates.

Nearly all of the indirect effect was through the risk factor pathway, while the indirect effects through stroke prevention drugs and the dependence between the mediators in the two pathways were small (Table 3).

When using alternative regression models only including main effects the results were similar, with a slightly larger adjusted total association and direct effect (S3 Table). Repeating the main analyses on singly imputed data also gave similar results (S4 Table).

## Discussion

We found that low SES, measured by low education, was associated with more severe strokes, where patients with low education were found to have 140 additional severe strokes per 10 000 patients over the study period compared to patients with mid/high education, adjusting for confounders. Almost 30% of this association was found to be attributable to an indirect effect via risk factors. Looking at the pathway we saw that diabetes, previous stroke and ADL-dependency were associated with both low education and more severe strokes. Differences in stroke prevention drugs could not explain the association between low SES and stroke severity.

The pathways between SES and outcomes after stroke are complex and here we have focused on the pathways that connect SES and stroke severity. To our knowledge this is the first study to investigate the extent to which risk factors and stroke prevention drugs contribute to the SES-stroke severity pathway.

Our results indicate that risk factors should be a target to decrease stroke severity among those with low SES. From a clinical point of view, we think that targeting diabetes would be beneficial in reducing the inequality in stroke severity among different levels of SES. Type 2 diabetes accounts for 85–90% of all diabetes in Sweden [29]. Lifestyle factors have a large impact on the risk of type 2 diabetes, a study found that 9 out of 10 new cases of diabetes in older adults could be attributable to the lifestyle factors obesity, physical activity, diet, smoking and alcohol intake [30]. It is unclear whether physical activity differs between levels of SES, while there is evidence that obesity, smoking, worse diet and more alcohol intake are associated with lower SES [31–34]. Hence, primary prevention of diabetes targeting these risk factors is desirable.

Regarding the influence of ADL-dependency on stroke severity, previous studies have shown that those with low SES are at greater risk of being ADL-dependent [35]. Behavioral risk factors and co-morbidity seem to play a major role in these inequalities [36]. An aim for future studies using mediation analysis would be to target why ADL-dependency is more prevalent among those with low SES.

A major strength of our study is that it covers an unselected population since all hospitals that care for patients with acute stroke in Sweden are included and that Riksstroke has been shown to have excellent coverage [19]. Data is also prospectively collected and regularly validated, which together with low levels of missing data vouch for high data quality [37].

Due to the computationally intensive estimation method, the use of multiple imputation was deemed infeasible, and a complete case analysis was therefore performed. The proportion of missing data in the study was relatively low, and sensitivity analysis using single imputation showed similar results. Missing data is unlikely to have impacted the results.

A drawback of observational studies in general and in our study is that residual confounding cannot be completely ruled out. For the methods used in this study we assume that there are no unobserved confounders of the mediator-outcome relationships, i.e., no unobserved confounders of the risk factor-stroke severity or stroke prevention drugs-stroke severity pathways. In our analyses we have adjusted for the baseline confounders age and sex as well as year of stroke to minimize any confounding temporal effects. By the retrospective design we are restricted by the predefined set of variables that are collected in the register, this affects the possibilities to adjust for confounding and also the potential mediators that can be included in the study. For example, there is no information regarding stroke volume and location, compliance to medication or laboratory tests, and information on medication with antihypertensives is provided as a proxy for information regarding if the patient has hypertension or not. Except for smoking, information on lifestyle factors is not registered in Riksstroke and was therefore not included in the risk factor pathway. It has been shown that a healthy diet, a physically active lifestyle and lower alcohol consumption are associated with less severe strokes [2, 38, 39]. It is therefore likely that the mediating role of risk factors is underestimated in our study. Future studies combining Riksstroke data with health surveys that collect a wider variety of lifestyle variables could shed further light on the role of risk factors in combination with SES.

We have used level of consciousness as a proxy for stroke severity. A variable with higher resolution would be NIHSS, but levels of missing data are >50% for this variable compared to 1.6% for level of consciousness. Rather than performing multiple imputation of NIHSS, which requires the additional unverifiable assumption that data are missing at random (MAR) [27], we opt for using the more complete variable. Level of consciousness has been shown to be a good proxy for NIHSS in predicting death after stroke [40].

We used education as proxy for SES. Education is associated with social status and may be related to both access to material and non-material resources such as knowledge that affects health behaviors, while income relates more directly to economic status and material resources [20]. An advantage to using education is that it is established early in life and is less variable across the lifespan compared to income, which is particularly important in an elderly population group. Furthermore, in the current study we used an individual measure of SES, but an area-based measure could add further insights into the importance of the context that a person inhabits [41, 42].

Different versions of direct and indirect effects have been suggested [23]. We have estimated so-called interventional disparity direct and indirect effects via risk factors and/or stroke prevention drugs [24]. These effect types have the advantage that path-specific effects can be estimated without having to assume that there are no unobserved common causes of the mediator variables, which is unlikely to be fulfilled in practice, and are also valid even when the true underlying direction of associations between the mediators are unknown [26]. By using a disparity focused approach we shift the focus from the effect of intervening on SES directly, which is difficult in practice, to more policy relevant questions regarding what would happen to the SES disparity if interventions on intermediate variables were implemented.

The estimation of the effects is based on regression models and the results therefore vary depending on how these are specified. To reduce the risk of model misspecification bias we specified flexible models including interactions and higher order terms. To check the robustness of our results these were compared to analyses based on simple models only including main effects and no substantial differences were found.

A large proportion of the association between low education and severe stroke was a direct effect, not operating through either the risk factor or stroke prevention drugs pathways. To shift the ratio from direct to indirect effects and hence offer more explanation of the relationships, the data resolution should be higher and include a larger set of possible factors of interest.

Finally, our study is set in Sweden where both education and health care are publicly financed. It is important to note that the generalizability of our findings may be restricted to countries with similar population demographics and welfare systems.

## Conclusion

We found that almost 30% of the increased risk of a more severe stroke among low SES patients stems from differences in risk factors, while the effect of stroke prevention drugs was negligible. Hence, risk factors should be addressed more aggressively by clinicians to decrease stroke severity in those with low education. We also hope to inspire a more widespread use of mediation analysis by showing the potential in elucidating complex relationships between SES and health outcomes.

## Supporting information

S1 Table The RECORD statement–Checklist of items, extended from the STROBE statement, that should be reported in observational studies using routinely collected health data. (DOCX)

S2 Table Patient characteristics, all patient records. Number (%).
(DOCX)

S3 Table Adjusted total association and direct, and indirect effects estimated as absolute risk differences (excess risks) based on models with main effects only. Estimates based on 200 Monte Carlo simulations and standard errors based on 1000 bootstrap replicates.
(DOCX)

S4 Table Adjusted total association and direct, and indirect effects estimated as absolute risk differences (excess risks) based on singly imputed data (stochastic imputation using chained equations with 10 burn in iterations). Estimates based on 500 Monte Carlo simulations.
(DOCX)

## References

[1] Collaborators GBDS. Global, regional, and national burden of stroke and its risk factors, 1990–2019: a systematic analysis for the Global Burden of Disease Study 2019. Lancet Neurol. 2021;20(10):795–820. doi: 10.1016/S1474-4422(21)00252-0 34487721 PMC8443449

[2] O’Donnell MJ, Chin SL, Rangarajan S, Xavier D, Liu L, Zhang H, et al. Global and regional effects of potentially modifiable risk factors associated with acute stroke in 32 countries (INTERSTROKE): a case-control study. Lancet. 2016;388(10046):761–75. doi: 10.1016/S0140-6736(16)30506-2 27431356

[3] Rosengren A, Smyth A, Rangarajan S, Ramasundarahettige C, Bangdiwala SI, AlHabib KF, et al. Socioeconomic status and risk of cardiovascular disease in 20 low-income, middle-income, and high-income countries: the Prospective Urban Rural Epidemiologic (PURE) study. Lancet Glob Health. 2019;7(6):e748–e60. doi: 10.1016/S2214-109X(19)30045-2 31028013

[4] Avan A, Digaleh H, Di Napoli M, Stranges S, Behrouz R, Shojaeianbabaei G, et al. Socioeconomic status and stroke incidence, prevalence, mortality, and worldwide burden: an ecological analysis from the Global Burden of Disease Study 2017. BMC Med. 2019;17(1):191. doi: 10.1186/s12916-019-1397-3 31647003 PMC6813111

[5] Winkleby MA, Jatulis DE, Frank E, Fortmann SP. Socioeconomic status and health: how education, income, and occupation contribute to risk factors for cardiovascular disease. Am J Public Health. 1992;82(6):816–20. doi: 10.2105/ajph.82.6.816 1585961 PMC1694190

[6] Kerr GD, Higgins P, Walters M, Ghosh SK, Wright F, Langhorne P, et al. Socioeconomic Status and Transient Ischaemic Attack/Stroke: A Prospective Observational Study. Cerebrovasc Dis. 2011;31(2):130–7. doi: 10.1159/000321732 21109736

[7] Arrich J, Müllner M, Lalouschek W, Greisenegger S, Crevenna R, Herkner H. Influence of Socioeconomic Status and Gender on Stroke Treatment and Diagnostics. Stroke. 2008;39(7):2066–72. doi: 10.1161/STROKEAHA.107.506147 18436881

[8] Rey V, Faouzi M, Huchmand-Zadeh M, Michel P. Stroke initial severity and outcome relative to insurance status in a universal health care system in Switzerland. Eur J Neurol. 2011;18(8):1094–7. doi: 10.1111/j.1468-1331.2010.03264.x 21749574

[9] Bray BD, Paley L, Hoffman A, James M, Gompertz P, Wolfe CDA, et al. Socioeconomic disparities in first stroke incidence, quality of care, and survival: a nationwide registry-based cohort study of 44 million adults in England. Lancet Public Health. 2018;3(4):e185–e93. doi: 10.1016/S2468-2667(18)30030-6 29550372 PMC5887080

[10] Lindmark A, Glader EL, Asplund K, Norrving B, Eriksson M. Socioeconomic disparities in stroke case fatality—Observations from Riks-Stroke, the Swedish stroke register. Int J Stroke. 2014;9(4):429–36. doi: 10.1111/ijs.12133 23981768

[11] Glader EL, Edlund H, Sukhova M, Asplund K, Norrving B, Eriksson M. Reduced inequality in access to stroke unit care over time: a 15-year follow-up of socioeconomic disparities in Sweden. Cerebrovasc Dis. 2013;36(5–6):407–11. doi: 10.1159/000355497 24247019

[12] Stecksén A, Glader E-L, Asplund K, Norrving B, Eriksson M. Education Level and Inequalities in Stroke Reperfusion Therapy. Stroke. 2014;45(9):2762–8. doi: 10.1161/STROKEAHA.114.005323 25074515

[13] Sjölander M, Eriksson M, Asplund K, Norrving B, Glader E-L. Socioeconomic Inequalities in the Prescription of Oral Anticoagulants in Stroke Patients With Atrial Fibrillation. Stroke. 2015;46(8):2220–5. doi: 10.1161/STROKEAHA.115.009718 26081841

[14] Kerr GD, Slavin H, Clark D, Coupar F, Langhorne P, Stott DJ. Do Vascular Risk Factors Explain the Association between Socioeconomic Status and Stroke Incidence: A Meta-Analysis. Cerebrovasc Dis. 2011;31(1):57–63. doi: 10.1159/000320855 20980755

[15] Marshall IJ, Wang Y, Crichton S, McKevitt C, Rudd AG, Wolfe CDA. The effects of socioeconomic status on stroke risk and outcomes. Lancet Neurol. 2015;14(12):1206–18. doi: 10.1016/S1474-4422(15)00200-8 26581971

[16] VanderWeele TJ. Explanation in causal inference: Methods for mediation and interaction. 1 ed. New York, NY: Oxford University Press; 2015.

[17] Kleindorfer D, Lindsell C, Alwell KA, Moomaw CJ, Woo D, Flaherty ML, et al. Patients living in impoverished areas have more severe ischemic strokes. Stroke. 2012;43(8):2055–9. doi: 10.1161/STROKEAHA.111.649608 22773557 PMC3432858

[18] Lindmark A, Norrving B, Eriksson M. Socioeconomic status and survival after stroke—using mediation and sensitivity analyses to assess the effect of stroke severity and unmeasured confounding. BMC Public Health. 2020;20(1):554. doi: 10.1186/s12889-020-08629-1 32334556 PMC7183587

[19] Riksstroke. Stroke och TIA, Riksstrokes årsrapport 2015 (annual report 2015). 2016.

[20] Kingston PW, Hubbard R, Lapp B, Schroeder P, Wilson J. Why education matters. Sociol Educ. 2003;76(1):53–70.

[21] Shrier I, Platt RW. Reducing bias through directed acyclic graphs. BMC Med Res Methodol. 2008;8(1):70. doi: 10.1186/1471-2288-8-70 18973665 PMC2601045

[22] Baron RM, Kenny DA. The Moderator Mediator Variable Distinction in Social Psychological-Research—Conceptual, Strategic, and Statistical Considerations. J Pers Soc Psychol. 1986;51(6):1173–82. doi: 10.1037//0022-3514.51.6.1173 3806354

[23] Nguyen TQ, Schmid I, Stuart EA. Clarifying causal mediation analysis for the applied researcher: Defining effects based on what we want to learn. Psychol Methods. 2020;26(2):255–71. 10.1037/met0000299 PMC8496983 32673039

[24] Micali N, Daniel RM, Ploubidis GB, De Stavola BL. Maternal Prepregnancy Weight Status and Adolescent Eating Disorder Behaviors: A Longitudinal Study of Risk Pathways. Epidemiology. 2018;29(4):579–89. doi: 10.1097/EDE.0000000000000850 29750675

[25] Moreno-Betancur M, Moran P, Becker D, Patton GC, Carlin JB. Mediation effects that emulate a target randomised trial: Simulation-based evaluation of ill-defined interventions on multiple mediators. Stat Methods Med Res. 2021;30(6):1395–412. doi: 10.1177/0962280221998409 33749386 PMC8371283

[26] Vansteelandt S, Daniel RM. Interventional effects for mediation analysis with multiple mediators. Epidemiology. 2017;28(2):258–65. doi: 10.1097/EDE.0000000000000596 27922534 PMC5289540

[27] White IR, Royston P, Wood AM. Multiple imputation using chained equations: Issues and guidance for practice. Stat Med. 2011;30(4):377–99. doi: 10.1002/sim.4067 21225900

[28] R Core Team. R: A language and environment for statistical computing. Vienna, Austria: R Foundation for Statistical Computing; 2021.

[29] Andersson T, Ahlbom A, Carlsson S. Diabetes Prevalence in Sweden at Present and Projections for Year 2050. PloS One. 2015;10(11). doi: 10.1371/journal.pone.0143084 26619340 PMC4664416

[30] Mozaffarian D, Kamineni A, Carnethon M, Djoussé L, Mukamal KJ, Siscovick D. Lifestyle risk factors and new-onset diabetes mellitus in older adults: the cardiovascular health study. Arch Intern Med. 2009;169(8):798–807. doi: 10.1001/archinternmed.2009.21 19398692 PMC2828342

[31] Stalsberg R, Pedersen AV. Are Differences in Physical Activity across Socioeconomic Groups Associated with Choice of Physical Activity Variables to Report? Int J Environ Res Public Health. 2018;15(5):922. doi: 10.3390/ijerph15050922 29734745 PMC5981961

[32] Pechey R, Monsivais P. Socioeconomic inequalities in the healthiness of food choices: Exploring the contributions of food expenditures. Prev Med. 2016;88:203–9. doi: 10.1016/j.ypmed.2016.04.012 27095324 PMC4910945

[33] Molarius A, Lindén-Boström M, Granström F, Karlsson J. Obesity continues to increase in the majority of the population in mid-Sweden—a 12-year follow-up. Eur J Public Health. 2016;26(4):622–7. doi: 10.1093/eurpub/ckw042 27074794

[34] Centralförbundet för alkohol- och narkotikaupplysning C. Socioekonomiska skillnader i beroende och utsatthet för andras användning av alkohol, narkotika och tobak. Stockholm 2018. Report No.: 176.

[35] Lima-Costa MF, Mambrini JVM, Peixoto SV, Malta DC, Macinko J. Socioeconomic inequalities in activities of daily living limitations and in the provision of informal and formal care for noninstitutionalized older Brazilians: National Health Survey, 2013. Int J Equity Health. 2016;15(1):137. doi: 10.1186/s12939-016-0429-2 27852307 PMC5112736

[36] Ramsay SE, Whincup PH, Morris RW, Lennon LT, Wannamethee SG. Extent of Social Inequalities in Disability in the Elderly: Results From a Population-based Study of British Men. Ann Epidemiol. 2008;18(12):896–903. doi: 10.1016/j.annepidem.2008.09.006 19041588 PMC2728204

[37] Soderholm A, Stegmayr B, Glader EL, Asplund K, Riksstroke C. Validation of Hospital Performance Measures of Acute Stroke Care Quality. Riksstroke, the Swedish Stroke Register. Neuroepidemiology. 2016;46(4):229–34. doi: 10.1159/000444679 26975057

[38] Reinholdsson M, Palstam A, Sunnerhagen KS. Prestroke physical activity could influence acute stroke severity (part of PAPSIGOT). Neurology. 2018;91(16):e1461–e7. doi: 10.1212/WNL.0000000000006354 30232251 PMC6202943

[39] Tsivgoulis G, Psaltopoulou T, Wadley VG, Alexandrov AV, Howard G, Unverzagt FW, et al. Adherence to a Mediterranean diet and prediction of incident stroke. Stroke. 2015;46(3):780–5. doi: 10.1161/STROKEAHA.114.007894 25628306 PMC4621211

[40] Bray BD, Campbell J, Cloud GC, Hoffman A, James M, Tyrrell PJ, et al. Derivation and external validation of a case mix model for the standardized reporting of 30-day stroke mortality rates. Stroke. 2014;45(11):3374–80. doi: 10.1161/STROKEAHA.114.006451 25293667

[41] Arcaya MC, Arcaya AL, Subramanian SV. Inequalities in health: definitions, concepts, and theories. Glob Health Action. 2015;8:27106. doi: 10.3402/gha.v8.27106 26112142 PMC4481045

[42] Kawachi I, Subramanian SV, Almeida-Filho N. A glossary for health inequalities. J Epidemiol Community Health. 2002;56(9):647–52. doi: 10.1136/jech.56.9.647 12177079 PMC1732240

