# Supplementary material for: Socioeconomic status and stroke severity: Understanding indirect effects via risk factors and stroke prevention using innovative statistical methods for mediation analysis
Source: PLoS One. 2022 Jun 24;17(6):e0270533. doi: 10.1371/journal.pone.0270533 (PMC9232158; doi:10.1371/journal.pone.0270533)
Supplement: S2 Table — Number (%). (DOCX) [file pone.0270533.s002.docx]

**S2 Table. Patient characteristics, all patient records. Number (%).**

| Variable | n | (%) |
| --- | --- | --- |
| *Exposure:* |  |  |
| Education |  |  |
| Low (primary school) | 43 778 | (43.2) |
| Mid/high (secondary school/university) | 55 489 | (54.8) |
| Missing | 1994 | (2.0) |
| *Outcome:* |  |  |
| Level of consciousness |  |  |
| Fully conscious | 86 358 | (85.3) |
| Lowered | 13 320 | (13.2) |
| Missing | 1583 | (1.6) |
| *Mediators:* |  |  |
| Smoker |  |  |
| Yes | 13 352 | (13.2) |
| No | 78 326 | (77.4) |
| Missing | 9583 | (9.5) |
| Diabetes |  |  |
| Yes | 22 117 | (21.8) |
| No | 78 622 | (77.6) |
| Missing | 522 | (0.5) |
| Atrial fibrillation |  |  |
| Yes | 30 196 | (29.8) |
| No | 70 253 | (69.4) |
| Missing | 812 | (0.8) |
| Previous stroke |  |  |
| Yes | 23 446 | (23.2) |
| No | 77 166 | (76.2) |
| Missing | 649 | (0.6) |
| ADL dependent at baseline |  |  |
| Yes | 12 536 | (12.4) |
| No | 85 649 | (84.6) |
| Missing | 3076 | (3.0) |
| Antihypertensives |  |  |
| Yes | 67 455 | (66.6) |
| No | 32 934 | (32.5) |
| Missing | 872 | (0.9) |
| Statins |  |  |
| Yes | 29 784 | (29.4) |
| No | 70 599 | (69.7) |
| Missing | 878 | (0.9) |
| Antiplatelets |  |  |
| Yes | 39 440 | (38.9) |
| No | 61 080 | (60.3) |
| Missing | 741 | (0.7) |
| Anticoagulants |  |  |
| Yes | 10375 | (10.2) |
| No | 90136 | (89.0) |
| Missing | 750 | (0.7) |
| *Confounders:* |  |  |
| Sex |  |  |
| Male | 52 719 | (52.1) |
| Female | 48 542 | (47.9) |
| Year of stroke |  |  |
| 2012 | 21 522 | (21.3) |
| 2013 | 20 945 | (20.7) |
| 2014 | 20 369 | (20.1) |
| 2015 | 19 704 | (19.5) |
| 2016 | 18 721 | (18.5) |
| Age |  |  |
| 18-54 | 6117 | (6.0) |
| 55-64 | 10 718 | (10.6) |
| 65-74 | 24 794 | (24.5) |
| 75-84 | 32 359 | (32.0) |
| 84+ | 27 273 | (26.9) |

ADL: Activities in daily living.
