# Supplementary material for: Socioeconomic status and stroke severity: Understanding indirect effects via risk factors and stroke prevention using innovative statistical methods for mediation analysis
Source: PLoS One. 2022 Jun 24;17(6):e0270533. doi: 10.1371/journal.pone.0270533 (PMC9232158; doi:10.1371/journal.pone.0270533)
Supplement: S3 Table — Estimates based on 200 Monte Carlo simulations and standard errors based on 1000 bootstrap replicates. (DOCX) [file pone.0270533.s003.docx]

**S3 Table.** **Adjusted total association and direct, and indirect effects estimated as absolute risk differences (excess risks) based on models with main effects only.**

| Effect | Absolute risk difference | 95% CI | P-value | % of Adj. total association |
| --- | --- | --- | --- | --- |
| **Adjusted total association** | 1.5% | (1.1%-1.9%) | <0.001 |  |
| **Direct** | 1.1% | (0.7%-1.6%) | <0.001 | 76.1 |
| **Indirect via** |  |  |  |  |
| **all mediators** | 0.4% | (0.3%-0.4%) | <0.001 | 23.9 |
| **risk factors** | 0.4% | (0.3%-0.5%) | <0.001 | 25.0 |
| **stroke prevention drugs** | -0.02% | (-0.04%-0.00%) | 0.120 | -1.2 |
| **dependence between risk factors and stroke prevention drugs** | 0.00% | (-0.00%-0.01%) | 0.418 | 0.1 |

Estimates are based on 200 Monte Carlo simulations and standard errors are based on 1000 bootstrap replicates.
