# Supplementary material for: Socioeconomic status and stroke severity: Understanding indirect effects via risk factors and stroke prevention using innovative statistical methods for mediation analysis
Source: PLoS One. 2022 Jun 24;17(6):e0270533. doi: 10.1371/journal.pone.0270533 (PMC9232158; doi:10.1371/journal.pone.0270533)
Supplement: S4 Table — Estimates based on 500 Monte Carlo simulations. (DOCX) [file pone.0270533.s004.docx]

**S4 Table.** **Adjusted total association and direct, and indirect effects estimated as absolute risk differences (excess risks) based on singly imputed data (stochastic imputation using chained equations with 10 burn in iterations).**

| Effect | Absolute risk difference | % of Adj. total association |
| --- | --- | --- |
| **Adjusted total association** | 1.5% |  |
| **Direct** | 1.1% | 70.3 |
| **Indirect via** |  |  |
| **all mediators** | 0.5% | 29.7 |
| **risk factors** | 0.5% | 29.6 |
| **stroke prevention drugs** | -0.02% | -1.3 |
| **dependence between risk factors and stroke prevention drugs** | 0.02% | 1.3 |

Estimates are based on 500 Monte Carlo simulations.
